# Life and Living in Advanced Age: A Cohort Study in New Zealand -Te Puāwaitanga o Nga Tapuwae Kia Ora Tonu, LiLACS NZ: Study protocol

**DOI:** 10.1186/1471-2318-12-33

**Published:** 2012-06-29

**Authors:** Karen J Hayman, Ngaire Kerse, Lorna Dyall, Mere Kepa, Ruth Teh, Carol Wham, Valerie Wright-St Clair, Janine Wiles, Sally Keeling, Martin J Connolly, Tim J Wilkinson, Simon Moyes, Joanna B Broad, Santosh Jatrana

**Affiliations:** 1 Department of General Practice and Primary Healthcare, University of Auckland, Private Bay, 92109, Auckland, New Zealand; 2 Te Kupenga Hauora, Department of Māori Studies, University of Auckland, Auckland, New Zealand; 3 Institute of Food, Nutrition and Human Health, Massey University, Auckland, New Zealand; 4 School of Rehabilitation & Occupation Studies, Auckland University of Technology, Auckland, New Zealand; 5 Department of Community Health, University of Auckland, Auckland, New Zealand; 6 Dept of Medicine, University of Otago, Christchurch, New Zealand; 7 Freemasons’ Department of Geriatric Medicine, University of Auckland, Auckland, New Zealand; 8 Alfred Deakin Research Institute, Deakin University, Sydney, Australia

**Keywords:** Advanced age, Successful ageing, Longitudinal study, Cohort, Indigenous health

## Abstract

**Background:**

The number of people of advanced age (85 years and older) is increasing and health systems may be challenged by increasing health-related needs. Recent overseas evidence suggests relatively high levels of wellbeing in this group, however little is known about people of advanced age, particularly the indigenous Māori, in Aotearoa, New Zealand. This paper outlines the methods of the study Life and Living in Advanced Age: A Cohort Study in New Zealand. The study aimed to establish predictors of successful advanced ageing and understand the relative importance of health, frailty, cultural, social & economic factors to successful ageing for Māori and non-Māori in New Zealand.

**Methods/design:**

A total population cohort study of those of advanced age. Two cohorts of equal size, Māori aged 80–90 and non-Māori aged 85, oversampling to enable sufficient power, were enrolled. A defined geographic region, living in the Bay of Plenty and Lakes District Health Board areas of New Zealand, defined the sampling frame. Rūnanga (Māori tribal organisations) and Primary Health Organisations were subcontracted to recruit on behalf of the University. *Measures -* a comprehensive interview schedule was piloted and administered by a trained interviewer using standardised techniques. Socio-demographic and personal history included tribal affiliation for Māori and participation in cultural practices; physical and psychological health status used standardised validated research tools; health behaviours included smoking, alcohol use and nutrition risk; and environmental data included local amenities, type of housing and neighbourhood. Social network structures and social support exchanges are recorded. Measures of physical function; gait speed, leg strength and balance, were completed. Everyday interests and activities, views on ageing and financial interests complete the interview. A physical assessment by a trained nurse included electrocardiograph, blood pressure, hearing and vision, anthropometric measures, respiratory function testing and blood samples.

**Discussion:**

A longitudinal study of people of advanced age is underway in New Zealand. The health status of a population based sample of older people will be established and predictors of successful ageing determined.

## Background

### The population is ageing

People over 85 years of age are the fastest growing population group in New Zealand. They currently form 1% of the population but by 2050 the proportion will have grown to 6% [1]. New Zealand men aged 83 and women aged 87 have a 90% chance of living another year (P90) [1]. Little is known about this group as the absolute numbers of people over age 85 typically included in routine surveys is small.

Expenditure on personal health and disability support for people in advanced age is the highest for any age group in New Zealand with an average yearly per capita expenditure of $13,640 for women and $12,144 for men [2]. This group also has the highest rate of preventable and unavoidable hospitalisations of any age group [3]. However, despite increasing frailty, chronic conditions and co-morbidities, the majority of those over age 85 are living independently in the community with or without assistance (70%: 2001 New Zealand Census), with women much more likely to be living alone than men [2,4-7]. New insights into associations with successful ageing in this group could lead to even small improvements in health and independence resulting in comparatively larger savings in health provision as the number of people over 85 years increases. Reallocation of current health funding may be prudent and better data will help inform where investment should be made and what the ongoing implications and opportunity costs would be. This paper describes the design of a longitudinal study to examine the contribution of a range of factors to independence and living well in advanced age. We begin by discussing current knowledge on the predictors of successful ageing both within and beyond New Zealand.

### Health for Māori living in advanced age

Māori are the indigenous people of Aotearoa, New Zealand, comprising 14% of the total population and 2% of those aged over 80 years [8]. Of those Māori who reach age 75, many have multiple health problems but may not have readily available whānau (extended family) to care for and support them due to the migration of whānau members from rural to urban areas, often for employment. Currently, there are few Māori who reach 85 years of age (less than 0.2% of the Māori population, [1]). Eighty to ninety years represents advanced age for Māori (P90 for Māori men is 79 years and for Māori women is 86 years). Thus, there is a large disparity in longevity for Māori and a disparity in their disability levels in advanced age. Demographic projections [9] suggest that Māori people can expect to live longer than current levels thereby potentially expanding the population of Māori living in advanced age and accentuating the disparities in disability.

Although Māori living in advanced age may enjoy wider social connections than non-Māori through the role they play in whānau and communities, their level of income is low and this is likely to affect their well-being. Investigation of the settlement of claims made to the Waitangi Tribunal (New Zealand’s commission of inquiry into claims by Māori over breaches in the promises made in New Zealand’s founding document, the Treaty of Waitangi) may reveal material benefits to Māori living in advanced age.

### Ageing in New Zealand

The older population in New Zealand will have some attributes similar to other populations and some attributes distinct to New Zealand. Life expectancy in New Zealand, for instance, is almost identical to the United Kingdom, but patterns of disability-free life expectancy differ between the two countries. Risk of fall-related injuries was six times higher in older people living in New Zealand but born in the United Kingdom compared with those born in New Zealand, controlling for other factors [10]. In New Zealand, 20% of those aged 85 and older are in residential care [3]. Deprivation has been studied but not in old age [11], and the “potential collateral health gain” from social contacts has been relatively neglected [12]. Income, family structure, economic circumstances, housing, social status and health will all be important to understand well-being and successful ageing for the very old in New Zealand [13], and housing and economic factors are integral to well-being. Incomes of older people are modest at best, particularly for Māori, and are least for those over age 65 years [14].

### What we know from epidemiological studies

Longitudinal studies provide insight into epidemiological factors contributing to successful ageing. There are extensive reports predicting survival, health and function in those aged over 65 years. At least eleven completed longitudinal studies of ageing and thirty seven ongoing studies of older people are known [15]. North American and United Kingdom studies have amassed evidence of disease states and smoking as shortening longevity, and physical activity as lengthening survival [16-18]. The importance of socioeconomic status is established [19] and more specific studies show the prevalence, course and impact of changing cognition in ageing [20]. Studies examining medical, psychological, social and economic factors demonstrate the importance of breadth in protective factors [21]. They have led to studies such as the Ageing Well - European Study on Adult Well-being examining the influence of cultural and health system related factors to well-being and survival. Poor nutritional status in older people is now a well-established negative prognostic indicator [22] as is inadequate food intake in community living older people [23]. Studies from Australasia consistently identify specific health, health behaviours and social factors as predicting longevity and wellbeing [24-26], describing health and social factors and their influences on wellbeing and survival. Previously in New Zealand, longitudinal studies have highlighted the association between disability and falls with mortality [27,28].

However, with advanced age, variability in all physiological and functional parameters increases [29]. There is good evidence that factors known to predict successful ageing for those from 65 years may act quite differently in the oldest old population. New Zealand data suggests that direct extrapolation from trajectories of people aged 65–85 to people aged 85 and over is likely to be inaccurate, for example, in patterns of living circumstances, reasons for hospital admission and even in survival [30]. Longitudinal studies of those aged 85 have emphasised the predictive nature of inflammation and vascular factors [31] and the Newcastle 85+ study, which is examining biomedical and clinical markers in detail [32] shows that in general people are independent at age 85 [7].

### Non-medical factors neglected

Disability levels are modulated by measures of social support [33] and social contact is an independent and equal predictor of mortality and perceived health [34-37]. However, few studies of social participation have evaluated the relationship in the oldest old and the direction of the relationship has been inconsistent. There is debate as to how well the ‘essential ingredient’ of social support has been measured [38]. Social networks, supports and contacts also impact utilisation of health services. A high level of family support can delay or prevent admission to institutions and use of formal supports [39]. However, the relationship between general well-being and social network type is inconsistent [40] and the relationship between social networks and health may be different for the very old [41].

Housing conditions influence both the physical and psychological well-being of the occupants. Inadequate living conditions lead to increased stress levels, social isolation, poor health and a higher risk of disease and injury [42]. Numerous factors are involved in the relationships between housing and health, from the structure and maintenance of the building and its location to elements in the lifestyle of the resident, such as tenure and size of household [43]. These factors underline the positive relationship between higher socio-economic status and health [44-46].

This study addresses the gap in information about predictors of successful advanced ageing for Māori and non-Māori living in New Zealand. By non-Māori we mean all people of an ethnicity other than Māori, including New Zealand and other European, people from the Pacific Islands, African, Chinese, other Asian and Indian. A longitudinal study, Life and Living in Advanced Age: A Cohort Study in New Zealand (LiLACS NZ) focuses on health in advanced age taking a broad approach to assessment of non-medical determinants of health. A feasibility project funded by the Health Research Council of New Zealand tested the procedures for recruitment and assessment of people of advanced age and found that it was possible to engage with, enrol and assess older Māori and non-Māori [47].

### Study aims

The study has four main aims, to: 1) establish the health status of people in advanced age, 2) establish predictors of successful advanced ageing for older Māori and non-Māori, 3) describe trajectories in function and transitions in care for those in advanced age, and 4) establish the relative importance of health, frailty, cultural, social & economic factors (and others) in predicting relevant outcomes.

## Methods/design

### Study design

A total population longitudinal cohort study of those of advanced age living in a defined geographical region in New Zealand will address the aims. Two cohorts of equal size, Māori aged 80–90 and non-Māori aged 85 have been enrolled to allow equal explanatory power for the main analyses. Annual follow-up will assess on-going health status and function. The first three years have been funded and subsequent assessments are planned for fifteen years, funding permitting. The Northern X Regional Ethics Committee of New Zealand granted ethical approval for the longitudinal study in December 2009 (NTX/09/09/088).

### Eligibility and recruitment

Potential participants were those born between 1 January and 31 December 1925 (aged 85 in 2010) for non-Māori, and between 1 January 1920 and 31 December 1930 (aged 80–90 in 2010) for Māori. Those meeting the age criteria and living within the Lakes or Bay of Plenty District Health Board (DHB) areas during the 2010 enrolment year were eligible. The study has two cohorts running in parallel, one with Māori participants only, the other enrolling participants of all other ethnicities.

Though recruitment rates were modest in the feasibility project, we demonstrated we could access independent older people. This meant the larger longitudinal study needed to make a comprehensive attempt to increase representativeness of participants i.e. inclusion of more disabled older people and those who were not well known to social and health agencies. Local publicity before fieldwork began and during the study involved speaking on local radio stations and at meetings of older people such as kaumātua (Māori elder) groups, community service agencies and at rest homes, preparing newspaper articles and placing posters and pamphlets in public places such as doctor's surgeries, pharmacies and shopping malls. Dedicated local consultation was used to engage Māori people in the project. A Rōpū Kaitiaki o tikanga Māori (governance group to protect the principles of proper conduct for Māori in research) was formed and included six Māori leaders related to the regions of the study.

After extensive consultation and regional meetings with tribal leaders and older people, seven local organisations were subcontracted by the University of Auckland to contact, recruit and enroll participants and to conduct interviews and health assessments. They comprise three Primary Health Organisations (PHOs; organisations that manage data and distribute funds for groups of General Practitioners), and four Māori Rūnunga (Māori tribal organisations). Four organisations enrolled both Māori and non-Māori participants, two, working under a joint subcontract, enrolled only Māori and one enrolled only non-Māori. Having local organisations driving recruitment and assessments enhanced engagement in the study and facilitates efficient flow through the study for participants.

Several sources were used to ascertain as complete a sample of eligible older people as possible; the New Zealand General and Māori electoral rolls, primary care databases through PHOs and General Practice (GP) databases. These lists were supplemented through whānau and community networks. Older people were approached and the study introduced by a person known to them where possible. If this was not possible, contact was made by their health provider or Māori iwi (tribal group) representative. They were forwarded written information, or contacted by telephone by a researcher or visited by the Māori groups, to explain the study in full. Kaupapa Māori methods (ways specific to Māori) were used to engage with Māori participants [48]. Written informed consent was obtained before interview.

### Measures

Study measures were collected in three phases: a structured face-to-face standardised questionnaire, a health assessment and blood test, and a brief review of general practice medical records for diagnosed medical conditions. Development of interview schedules and examination processes are described elsewhere [47]. Interviews and assessments were offered as home visits or at another site as the participant chose. Sites of interview were expected to vary according to each local organisation’s preferred practice.

During the feasibility project focus groups with Māori and key informants discussed the topic of “living long and well in today’s society”. The main themes raised were formulated into questions about engagement and practice of Māori culture. The questions were refined by the Rōpū Kaitiaki and integrated into the main interview, with some questions to be asked of Māori only and some to be asked of both cohorts. Interview questions were translated for use in a bilingual version as needed.

**Figure 1:**
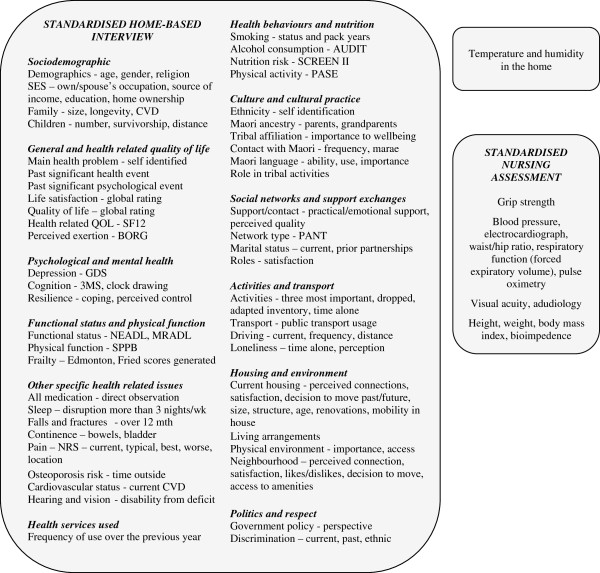
**The main areas of interview and physical assessment.** CVD – cardiovascular disease, QOL – quality of life, SF-12 – Medical Outcomes Study Short Form Health Survey (12 item), BORG – Borg Rating of Perceived Exertion Scale, GDS-15 – Geriatric Depression Scale (15 item), 3MS – Modified Mini Mental State Examination, NEADL - Nottingham Extended Activities of Daily Living Scale, MRADL - Manchester Respiratory Activities of Daily Living Scale, SPPB – Short Physical Performance Battery, NRS – Numerical Rating Scale, AUDIT – Alcohol Use Disorders Identification Test, SCREEN II - Seniors in the Community: Risk Evaluation for Eating and Nutrition (Version II), PASE – Physical Activity Scale for the Elderly, PANT - Practitioner Assessment of Network Type.

The comprehensive interview, conducted by a trained lay interviewer, took between two and four hours to complete excluding the consenting process. The factors measured in detail at baseline by interview were grouped into socio-demographic characteristics, general health and health related quality of life measures, psychological and mental health factors, functional status and physical function, other specific health-related issues, health behaviours including nutrition, health services used, culture and cultural practices, social networks and support exchanges, activities and transport, housing and environment and politics and respect. These are reported in detail and are shown in Figure 1.

#### Socio-demographic characteristics

We expanded standard demographic enquiry (age, ethnicity and current marital status) to include the length of time participants had been widowed/separated/divorced and the duration of other important relationships. We used an adapted version of the 2006 New Zealand Census questions for the highest education level achieved [49]. Past and current paid and voluntary work was queried. Standard of living was asked by using part of the Health Work and Retirement Study/New Zealand Longitudinal Study of Ageing (NZLSA) questionnaire [50]. Food security questions were included from the New Zealand National Nutrition Survey of 1997 [51] and childhood food deprivation was recorded. Family make up and survivorship was recorded after specifying “*Who raised you?*” For Māori, *whangai* is when a child is given to a couple who have no children to raise, similar to an open adoption. The age of biological parents at death was recorded if known and the number of brothers, sisters, sons and daughters ever and currently alive was asked including age at death or current age. Exact questions were adapted from the Newcastle 85+ study protocols [32].

#### General health and health related quality of life

General health status and health related quality of life was assessed with the Medical Outcomes Study Short Form Health Survey (SF 12) [52-54]. Self-rated health compared with others of the same age was also asked. Two questions from the 2006 New Zealand Census asked about disability arising from a health condition [49]. Two questions from the NZLSA questionnaire measured global life satisfaction and quality of life [50]. Past significant health and psychological events were recorded following two questions “*Have you ever had a major injury or health event that has affected you in the long term?*” and “*Have you ever had a major psychological stress event that has affected you in the long term?*” Answers were recorded verbatim for later coding. Cardiovascular disease (CVD) was recorded by self-report from a list of standard diagnoses and family history of CVD was coded using items from the Cardiovascular Health Study [55]. Items from the Manchester Respiratory Activities of Daily Living Scale [56] were added to the measure of functional status (below) to assess disability related to respiratory disease. The Borg Rating of Perceived Exertion Scale [57,58] was used to assess breathlessness at time of rest and after exertion.

#### Psychological and mental health

Cognition was assessed using the modified Mini-Mental State Examination (3MS) [59] and the clock drawing test [60], with depressive symptoms assessed using the Geriatric Depression Scale (GDS-15) [61]. Mastery and sense of control were assessed using the Pearlin and Schooler Sense of Mastery Scale [62]. Five original items were prepared for the study to assess perceived coping ability. Participants rated the question “*Thinking about .... how well do you cope?*” in regard to a) life overall, b) times of loss, c) financial hardship, d) on-going health problems and e) family troubles. Responses on a five-point Likert scale ranged from ‘not at all’ to ‘extremely’ well.

#### Functional status and physical function

Function was measured by direct observation; by timed walking speed, leg strength and balance using the Short Physical Performance Battery (SPPB) [63]. The Nottingham Extended Activities of Daily Living (NEADL) Scale [64] was used to assess self-reported functional status. Additional activities of daily living items, added in the same format as the NEADL, included grooming, toileting, transferring in and out of bed, showering/bathing and dressing. Two frailty measures were included. The Fried frailty scale [65] and the Edmonton Frail Scale [66] were able to be constructed from interview items.

#### Other specific health related issues

All medication (prescribed, over the counter, supplements and vitamins) were viewed by the trained interviewers and recorded by generic name as seen on the bottles and packets. Non-adherence was assessed by the question *“At times do you forget to take your prescription medication?”* and responses spanned four categories ranging from ‘all the time’ to ‘never’. Trouble with sleep quality was assessed with a positive response at least three nights a week such that it *“interferes with your activities the following day” *[67]. We asked whether sleep problems were present when participants were younger.

Participants were asked if they had fallen or sustained fractures over the last 12 months and one question assessed confidence in completing daily activities without falling. The Study of Osteoporotic Fractures osteoporosis screening tool was used to establish fracture risk [68]. Urinary and faecal continence were each assessed with a question about losing control of urine/bowels. One question sought to determine how much of a problem urinary incontinence was.

Pain was assessed using a numerical pain rating scale ranging from 0-no pain to 10-worst possible pain, and assessing current and typical pain and pain at its best and worst. Pain drawings are accepted assessment tools for chronic [69] and acute [70] pain and so participants were also asked to locate the site(s) of identified pain on a pain diagram. Disability caused by poor hearing and vision was assessed using modified questions from the Cognitive Function and Ageing Studies [71] i.e. “*How much does your hearing [vision] interfere with normal day-to-day functioning?*”. Hearing aid use and self-reported causes of visual impairment were recorded.

#### Health behaviours including nutrition

Smoking status was asked in a series of questions, ever smoked, when started, when stopped, how many cigarettes on average, to enable a pack year history to be calculated. The first two questions of the Alcohol Use Disorders Identification Test (AUDIT) [72] were used to establish alcohol use. Nutrition risk was determined using the 14-item validated questionnaire SCREEN II (Seniors in the Community: Risk Evaluation for Eating and Nutrition, Version II). This provides information on weight change, food intake and risk factors for food intake (meal frequency, diet restriction, appetite, chewing and swallowing difficulties, meal replacement, eating alone, meal preparation and shopping difficulties). Items are scored and summed. A cut-off of less than 50 (out of a possible 64) is considered to identify significant nutrition risk [73,74]. Whether participants had dentures or their own teeth and a reason for difficulties chewing, if any, supplemented the nutrition risk items. Physical activity was assessed with the Physical Activity Scale for the Elderly (PASE) validated in community-dwelling older adults [75]. PASE consists of ten items used to identify leisure, household and occupational related activity, and the duration of each activity over a one-week period.

#### Health services used

An inventory of primary and secondary health care providers was compiled for the feasibility project and modified in the main study; respondents were asked to recall the frequency of use over the last year.

#### Culture and cultural practices

Ethnicity was ascertained by self-identification, including languages spoken, hapū (wider family group), iwi (tribal group) and rohe (iwi boundary area) [49]. Questions about Māori ancestry included whether parents and grandparents were born Māori or lived as Māori. Questions used in the NZLSA Study and adapted from the Te Hoa Nuku Roa [76] scale of cultural identity were used to assess level of contact with Māori culture, including marae visits and contact with Māori people. Other questions about cultural activities were generated from discussion groups with older Māori and included: roles within the whānau, community and Māori society and satisfaction with those roles; the importance of hapū and iwi to wellbeing and the understanding of tikanga (cultural practices); special foods that are important to practising culture; the importance of nature and the outdoors; and whether participants were living in the same area as their hapū. The use of te reo Māori me ona tikanga (Māori language) or other non-English language was recorded and questions followed to find out where these languages were spoken and whether and how often the participant sought out opportunities to listen to the language. The importance of language/culture and values to wellbeing was asked with a five point categorical response scale ranging from ‘not at all’ to ‘extremely’ important. Religious affiliation was recorded and the importance of faith to wellbeing via a similar scale [49].

#### Social networks and support exchanges

The MacArthur Studies of Successful Ageing [77] questions were used as a base to measure availability of emotional and practical support. Wenger’s Network Assessment Instrument was used to establish the type of support network [78,79]. Participants’ satisfaction with the contact they had with family and friends was asked by adapting questions from the Duke Social Support Index [80].

The frequency of receiving meal services, home help, help for personal care and other services as used on a weekly basis was questioned and the funding source recorded.

#### Activities and transport

“*Of all the things you do, which three would you say are most important to you?*” was asked, adapted from the Melbourne Longitudinal Studies on Healthy Ageing Program (MELSHA) [81], and for each, the frequency of engaging in that activity was recorded. Change in activity over the last five years was asked, particularly which activities had been dropped [82]. Nine activities from the Enhancing Wellbeing in an Ageing Society (EWAS) Study [83] were used to record activities and the frequency of participation in them over the last four weeks. An adaptation of the Modified NPS Interest Checklist [84] provided an additional eight clusters of activities and used the same response format. The perception of spending time was asked with two questions: “*Thinking of how you spend your time would you say “most days I…*” with responses being ‘don’t have enough to do’, ‘just keep busy enough’, and ‘always have more than enough to do’. Spending time alone was asked about with four categories to choose from, ranging from ‘always alone’ to ‘never alone’. Feelings of loneliness were asked about with a similar spread of responses; ‘always feel lonely’ to ‘never feel lonely’. Questions about driving, being driven, use of public transport and satisfaction with getting around, examined transport for older people.

#### Housing and environment

Questions about housing, neighbourhood and the environment were developed from interviews with older people in the control arm of the DeLLITE Trial [85] analysed with respect to place and space [86]. Connection to current place, neighbourhood and community was asked with a five-category Likert scale ranging from ‘not at all’ to ‘extremely’ connected. Pets were counted. Housing type, ownership, size and age were recorded with structured questions adapted from the English Longitudinal Study of Ageing [87]. Satisfaction with the home and its warmth in winter was assessed with a five-level response ranging from ‘very satisfied’ to ‘very dissatisfied’. A Brannan Mini Twin Dial meter was used at home interviews to record the ambient temperature and humidity of the room the participant spent most time in. Problems moving around inside the house were asked about via a menu of responses. Questions about renovations included the age of completed renovations and future desired renovations, including reasons for not completing these to date. The likelihood and enthusiasm of participants for moving in the future were asked about with a five- category response set ranging from ‘not at all’ to ‘extremely’ likely/enthusiastic. Why participants chose the home they were in was asked and a menu of reasons offered. We asked whether participants ‘liked’ their home and their neighbourhood and then specifically what they liked most and least about the neighbourhood from a menu including the ‘land/physical environment’, ‘amenities’, ‘age of housing/architecture’, ‘diversity/age/friendliness of people’, ‘length of time have lived here’ and ‘other reasons’. Reasons for choosing the neighbourhood was also asked about with a menu of choices including: ‘to be near or with children’, ‘to be near or with other relative’, ‘leisure activities’, ‘closer to health services and amenities’, ‘close to marae’, ‘returning to family land’, ‘climate/weather’ and ‘other reason’. Difficulty getting to the shops and amenities was asked about with a set menu of responses. The importance of nature and the outdoors for a) wellbeing, b) recreation and c) children/grandchildren was asked.

#### Politics and respect

Participants were asked about their views on the New Zealand Government’s policy with several questions worded similarly: “*In general how happy are you with the current Government policy on .....*” Responses ranged on a five point scale from ‘very happy’ to ‘very unhappy’. The areas of interest included Government policy on social services for older people, health services for older people, transport options for older people, the economy and race relations. Questions about the respect participants felt others gave them were asked to elicit older peoples’ views of their place in society and autonomy in making decisions. Questions were taken from the 2002/2003 New Zealand Health Survey [88]. Finally general perspectives on growing older were asked and qualitative responses recorded verbatim.

For participants with advanced disability whose families and doctors were unwilling to burden them with full participation an ‘essential information only’ option was offered. This brief questionnaire was substantially smaller than the main questionnaire and took about thirty minutes to complete and included age, gender, living arrangement, functional status and, if any, main cause of disability.

#### Health assessment

The LiLACS NZ health assessment followed the baseline interview, generally on a different day. The assessment took about sixty to ninety minutes to complete and was conducted by a study nurse (registered with the New Zealand Nursing Council) using standardised procedures. All equipment was portable. Blood pressure was taken with the validated Microlife A100 Plus automated blood pressure monitor; upper arm lying and standing readings were repeated three times and the arm used was recorded. Weight was recorded using the Tanita Innerscan Body Composition Monitor, BC-545. The scale also provided measures of body fat mass, muscle mass and total body water estimated by bioimpedance. Although this is not the most accurate measurement for body composition, accessibility to more sophisticated measures such as a computerised topography or DEXA (Dual-emission X-ray absorptiometry) scan was not feasible in this study. Pulse oximetry was completed using the SP5500 finger pulse oximeter. Hearing (without hearing aids) was assessed using the H3SD Universal Hearing Screener and recorded as hertz heard at 500, 1000, 2000 and 4000 hertz in each ear. Visual acuity was assessed with research standard ETDRS visual acuity charts, a distance vision chart placed at 3 metres and a near vision chart tested at a comfortable focal length (usually 40 cm; distance recorded) with the use of glasses for either test also recorded. In accordance with research recommendations the minimum level of illumination was set at 350 units of lux (an international unit of light emittance). Lux readings were recorded. Anthropometric measures followed the protocol advised by the National Nutrition Survey of New Zealand 1997 [51]. Height was measured twice with the SECA 213 free-standing stadiometer and a third time if the difference between the first two was more than 1centimetre. Waist and hip circumference were measured twice with a non-stretchable expandable tape and if the difference was more than 1 centimetre they were measured a third time. Muscle strength was assessed by measuring grip strength in both hands in the standing position or sitting if unable to stand, using the Takei digital handgrip dynamometer-Grip D. An electrocardiograph (ECG) was taken using the Welch Allyn CP200 12 lead ECG monitor. Forced vital lung capacity and forced expiratory volume were assessed on the CP200 monitor using the spirometry add-on.

#### Blood tests

Blood tests, taken after an overnight fast, were drawn by the study nurse or the local laboratory service. Analyses are planned to include the following however funds for analysis are not yet assured. Serum is stored securely at −80 °C.

· Inflammatory markers - fibrinogen, high sensitivity C-reactive protein, interleukin-6, tumour necrosis factor-alpha, erythrocyte sedimentation rate

· Lipid profile - total cholesterol, high-density lipoprotein cholesterol, low-density lipoprotein cholesterol and triglycerides

· Nutritional markers - insulin-like growth factor-1, albumin, globulin, vitamin B_12_, vitamin B_6_, zinc, copper

· Endocrine function - thyroid stimulating hormone, triiothyronine, thyroxine, testosterone (men only), parathyroid hormone, adjusted calcium, glucose, glycated hemoglobin, sex hormone binding globulin (insulin resistance)

· Haematologic function - full blood count, total iron binding capacity, serum iron, iron saturation, red blood cell folate

· Renal function

· Vitamin D

· Liver function tests

· Cardiac markers - brain natriuretic peptide

#### Medical records

With permission, existing medical diagnoses and procedures were accessed from the participant’s general practice patient records using their individual National Health Index (NHI) number. Study nurses or general practice staff recorded the presence of fourteen specified medical conditions and eight diagnostic or medical procedures and, where possible, the date they were first noted.

### On-going data collection

Participants will be contacted annually for follow-up assessments which will include an interview and health assessment. Hospitalisation and mortality outcomes data will be obtained by matching the NHI with New Zealand Health Information Services (NZHIS) and DHB data. This will be completed each year after enrolment until the participant’s death. Specific permission is requested for this in the consent process.

### Study timeline and procedure

#### Staff training and supervision

**Figure 2:**
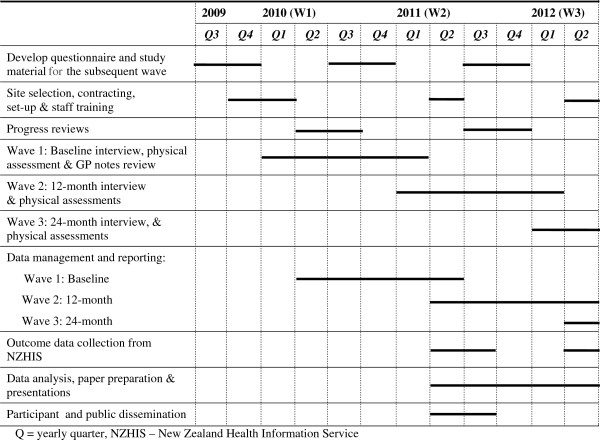
Timeline from inception to the end of current funding (Wave 3).

Figure 2 shows the timing of study procedures from inception to the end of current funding (Wave 3). Local organisations selected interviewers and nurses for the study, employing both Māori and non- Māori staff. In March 2010 the University of Auckland conducted a three day training programme, held in the region of the study, to teach standardised interview techniques and research protocols. All interviewers (nineteen) and nurses (eleven) attended. The training provided an overview of the study background and objectives and discussion of eligibility criteria and recruitment methods. Interviewers were provided full instruction on interview technique and ways to build rapport with older participants. Methods to ensure the safety of participants and staff during interviews were discussed and documented. Question guidelines were provided and discussed and interviewers had the opportunity during the training to practice asking questions with older volunteers.

A full set of equipment was provided for nurses to learn and practice on in pairs during the training sessions and to use for LiLAC Study assessments afterwards. Because spirometry testing poses some risks with older people, specific instruction was conducted by a spirometry specialist. Most study nurses were already trained in phlebotomy, however, phlebotomy procedures were taught locally by pathology laboratory trainers to those who needed it. Detailed procedure manuals and resources, including equipment manuals and usage guidelines for nurses, were provided for staff to keep for reference throughout the data collection phases.

#### Quality monitoring and data entry

Inter-rater reliability for interviewers was attained during initial interviews when they were paired and both recorded answers. Responses were reviewed and discrepancies discussed with coordinators to ensure consistency in interviewing technique. Two Auckland-based project managers supported field staff in the first year of data collection. Monthly site visits were arranged to meet with staff to discuss problems and difficulties in recruitment and data collection; to ensure standardised practices were maintained and update training as necessary; and to collect completed raw data. In addition to these monthly visits, two formal review meetings (June and September 2010) provided the opportunity for review of procedures and discussions between local and university staff.

In general, data was recorded manually on paper data forms. This method was chosen because of the difficulties of ensuring robust transport of electronic data from seven sites to one overall database and of providing appropriate technological support to outlying areas. All data forms were personally collected and returned to the study base in Auckland. ECG and spirometry tests were recorded electronically and an electronic form was also available to record medical conditions at the general practice. Data were logged in Auckland into a Microsoft Access database.

All data forms were checked thoroughly for missing codes or indistinct writing and generated queries were emailed to local study coordinators. Responses were changed manually on the data collection form before data entry. ECG and spirometry tests were read after administration in a standard manner by a cardiologist and physician. Test results were forwarded to the participant’s GP.

#### Ensuring participant safety

Adverse events, although unlikely during home visits, would not have the same back up as in a normal health care setting. If adverse events occurred during the interview the interviewer was instructed to respond as any lay person would and contact existing health providers and emergency services. Nurses were all trained clinicians and able to use their experience and judgement to contact the appropriate services should an emergency arise. The study protocols included information and emergency phone numbers if needed and a guide for when to alert GPs or emergency services. Study staff were encouraged to keep in contact with each other and each site met regularly to discuss concerns. The team of researchers based in Auckland were available by phone and email to provide back up and advice to the field staff and the participant. The local DHBs were supportive of the study and developed referral processes for any uncovered health needs. These were initiated through referral from the usual GP (with the older person’s permission) and only if information uncovered was clinically relevant and urgent.

### Sample power

There is an expectation of 20% mortality after two years. From the feasibility data several estimations are possible. At a rate of exposure of 23%, an NEADL score below 15 at baseline has the power to detect a relative risk of 1.67 if there are 500 persons in the sample. For a SCREEN II score of less than 50 to be related to a 1.6 relative risk of mortality, 450 persons are required. For other continuous measures a smaller number of persons are required. It is therefore possible that a cohort of 500 people will yield sufficient power to detect meaningful determinants of successful ageing and change in function and mood. Other cohorts have been of similar size (The Leiden 85+ study 599; Newcastle 85+ study 800).

We expect that some determinants of healthy ageing may differ between Māori and non-Māori and this together with lower life expectancy means that to achieve sufficient explanatory power for Māori a specifically Māori cohort was needed separate from non-Māori. For Māori who are aged 85, data that are comparable will be included in both cohorts. In addition this number will enable exact descriptions of health and social status for these vulnerable groups.

### Data analysis

Recruitment and non-response will be reported and non-response weights calculated separately for each cohort with consideration to age (in the Māori cohort), gender, rural/urban residence and service subcontractor using established survey techniques. These weights will be used to adjust estimates and confidence intervals in descriptive statistics. Their use in analyses of longitudinal data will be determined for each research question.

Descriptive statistics including proportions with 95% confidence intervals, means with standard deviations and medians will describe the health, economic, social and psychological status of the two cohorts at baseline. The extent of missing data will be reported. Regression analyses will be used to investigate differences between important subgroups.

Analyses of the longitudinal data such as mood and function which will be collected as the cohort progresses require the use of statistical techniques that allow the correlated nature of the data to be modelled. Generalised linear mixed models are an appropriate statistical method and, depending on the specifications within the model, can be used for normally distributed data (linear mixed models), binary, ordinal or categorical data (nonlinear mixed models) to investigate changes over time and the moderation of relationships by other factors over time. Cox’s proportional hazards models and extensions of these models for time dependent predictors will be used to investigate mortality. Models will include appropriate demographics, potential confounders and hypothesised risk factors and in particular, the effect of age on associations between outcomes and determinants will be investigated in the Māori cohort. The statistical packages SAS v9.2 and STATA v9 will be the main tools used in statistical analysis.

## Discussion

A comprehensive longitudinal study of people of advanced age is underway in New Zealand. This paper has outlined the methods of the study Life and Living in Advanced Age: A Cohort Study in New Zealand. The health status of a population based sample of older people has been established and over time predictors of successful ageing will be evaluated. Data will be able to be compared with those generated from other international longitudinal studies of ageing. Longevity is increasing in both Māori and non-Māori people, but remains lower in Māori. The narrow age band for non-Māori will reduce the variability in outcomes related to age alone, however a wider cohort age was sought for Māori as they represent a smaller proportion of New Zealand’s older population. Including a cohort of Māori will provide unique data for understanding the trajectories of ageing in New Zealand’s indigenous population.

Baseline data are collected by seven local organisations. The same organisations are contracted to undertake annual follow-up waves of data collection. Withdrawal due to increasing frailty is expected as a consequence of ageing but we believe that consistency in face to face contacts will encourage ongoing involvement in the study. In addition, relationships are built between participants and local study staff and are able to maximise participants’ satisfaction with study processes.

Participant time commitment is substantial and is a potential limitation to high enrolment numbers but we endeavor to enhance the experience of older participants by providing thank you cards following each data collection end and a toll free telephone number to contact the Auckland team at any time. Throughout the study flexibility is demonstrated in the approach to engaging with and retaining frail older participants [89]. We will host annual feedback meetings and provide written summaries to disseminate ongoing findings.

## Competing Interests

The author(s) declare that they have no competing interests.

## Authors’ contributions

KH contributed to the design of the study and provided project management oversight, NK conceived of the study and led its design and the development of outcome measures, LD and MK provided Māori leadership for the study, MK provided project management oversight, RT, CW, VWSC, JW, SK, MC, TW, JBB and SJ were involved in refining the study protocol and outcome measures. MC read and reported on spirometry tests. SM provided statistical advice. KH, NK, LD, MK, RT, CW, VWSC, SK, MC, TW, JBB participated in manuscript preparation. All authors read and approved the final manuscript.

## Pre-publication history

The pre-publication history for this paper can be accessed here:

http://www.biomedcentral.com/1471-2318/12/33/prepub
