# Erratum to: Life and living in advanced age: a cohort study in New Zealand -Te Puāwaitanga o Nga Tapuwae Kia Ora Tonu, LiLACS NZ: study protocol

**DOI:** 10.1186/s12877-017-0517-1

**Published:** 2017-06-19

**Authors:** Karen J Hayman, Ngaire Kerse, Lorna Dyall, Mere Kepa, Ruth Teh, Carol Wham, Valerie Wright-St Clair, Janine Wiles, Sally Keeling, Martin J Connolly, Tim J Wilkinson, Simon Moyes, Joanna B Broad, Santosh Jatrana

**Affiliations:** 1 Department of General Practice and Primary Healthcare, University of Auckland, Private Bay, Auckland, 92109 New Zealand; 2 Te Kupenga Hauora, Department of Māori Studies, University of Auckland, Auckland, New Zealand; 3 Institute of Food, Nutrition and Human Health, Massey University, Auckland, New Zealand; 4 School of Rehabilitation & Occupation Studies, Auckland University of Technology, Auckland, New Zealand; 5 Department of Community Health, University of Auckland, Auckland, New Zealand; 6 Dept of Medicine, University of Otago, Christchurch, New Zealand; 7 Freemasons’ Department of Geriatric Medicine, University of Auckland, Auckland, New Zealand; 8 Alfred Deakin Research Institute, Deakin University, Sydney, Australia

## Erratum

After the publication of this article [1] it was noted that the article PDF included reference to the article as part of the journal Aquatic Biosystems.

This affected:

1. An incorrect heading at the top of each page: Hayman et al. *Aquatic Biosystems* 2012, 12:33, which should be: Hayman et al. *BMC Geriatrics*, 2012, 12:33.

2. The citation at the end of the article which was:

Hayman et al.: Life and Living in Advanced Age: A Cohort Study in New Zealand -Te Puāwaitanga o Nga Tapuwae Kia Ora Tonu, LiLACS NZ: Study protocol. Aquatic Biosystems 2012 12:33.

This was incorrect and should be:

Hayman et al.: Life and Living in Advanced Age: A Cohort Study in New Zealand -Te Puāwaitanga o Nga Tapuwae Kia Ora Tonu, LiLACS NZ: Study protocol. BMC Geriatrics 2012 12:33.
